# Supplementary material for: Identification of photoperiod-induced specific miRNAs in the adrenal glands of Sunite sheep (Ovis aries)
Source: Front Vet Sci. 2022 Jul 22;9:888207. doi: 10.3389/fvets.2022.888207 (PMC9354845; doi:10.3389/fvets.2022.888207)
Supplement: Supplementary file 1 [file Data_Sheet_1.ZIP › Supplementary file/miRDeep2 file.zip!/miRDeep2 file/Novel_Structure/SP42A3/Novel_317.pdf]

Provisional ID	:	NC_019460.2_6589
Score total	:	1.4
Score for star read(s)	:	-1.3
Score for read counts	:	0
Score for mfe	:	1.1
Score for randfold	:	1.6
Score for cons. seed	:	
Total read count	:	96
Mature read count	:	96
Loop read count	:	0
Star read count	:	0

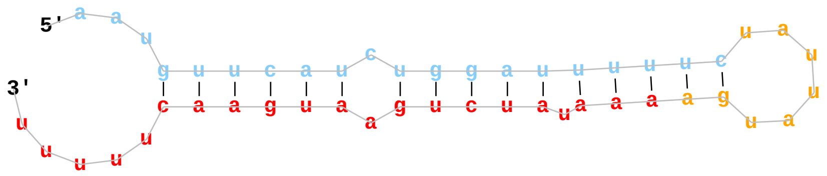

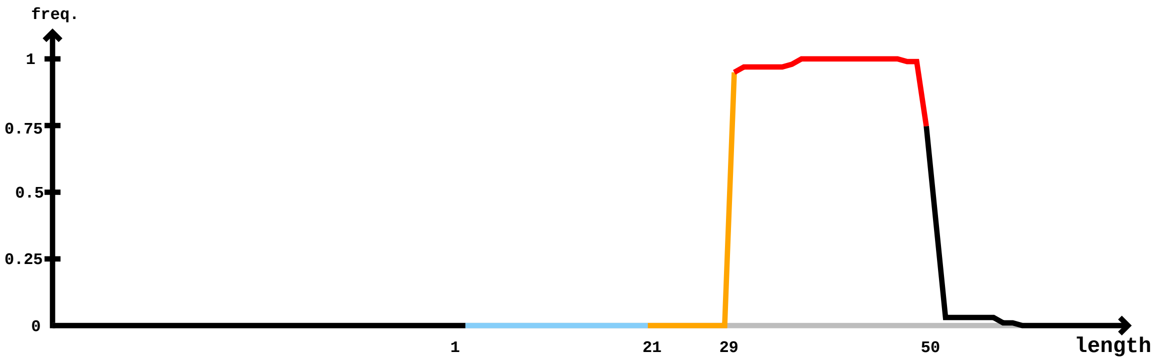

Star Mature

```
5'- aagaaauauuuucaaaaauauuuuaauuuuaaguuggccaaaauguucaucuggauuuuucuauuaugaaaauaucugaaugaacuuuuuggccaaucaaauacagguuu   -3'    exp
    ..(((((((((....)))))))))........((((((((((.((((((.((((((((((......))))).))))).)))))).))))))))))............   reads     mm        sample
    .....................................................................aaaAaucugaaugaacuu.......................   1         1         seq
    .....................................................................aaaAaucugaaugaacuuuu.....................   24        1         seq
    .....................................................................aaaAaucugaaugaacuuuuu....................   69        1         seq
    ......................................................................aauaucugaaugaacuuuuu....................   2         0         seq
    ............................................................................cugaaugaacuuuuuggccaau............   1         0         seq
    .............................................................................ugaaugaacuuuCuggccaau............   1         1         seq
    .............................................................................ugaaugaacuuuuuggccaaCca..........   1         1         seq
```
